# Alumni Association, University of Buffalo

**Published:** 1902-07

**Authors:** T. H. McKee

**Affiliations:** Secretary


### Alumni Association, University of Buffalo.

Reported by T. H. McKEE, M. D., Secretary.

The twenty-seventh annual meeting of the association was called to order at 2.30 o'clock, p.m., by first vice-president, A. W. Bayliss, who read a letter from the president, Dr. F. H. Moyer, explaining his absence on account of illness. The secretary was directed to write Dr. Moyer expressing the regrets and disappointments of the association at his absence, and hopes for his speedy recovery. A telegram of regret was also read from Dr. D. W. Harrington.

The report of the treasurer was presented and submitted to an auditing committee, consisting of Drs. Clark, Meisberger and B. G. Long, who reported the books and accounts correct. The report of committee was adopted.

Dr. A. T. Lytle, chairman, made a verbal report on the work of the executive committee, and moved a vote of thanks to Miss Chappell for her ever ready and cheerful assistance during the past year. Carried.

On motion the chairman was instructed to appoint a committee to nominate officers. The committee reported as follows: President, A. W. Bayliss, '89, Buffalo; first vice-president, A. W. Henchell, '89, Rochester; second vice-president, Fridolin Thoma, '87, Buffalo; third vice-president, Henry T. Benham, '90, Honeoye Falls; fourth vice-president, Jane W. Carroll, '91, Buffalo; fifth vice-president, Geo. A. Himmelsbach, '91, Buffalo; secretary, T. H. McKee, '98, Buffalo; treasurer, Herman K. DeGroat, '97, Buffalo.

*Executive Committee.*—Albert T. Lytle, '93, chairman, Buffalo; Grover W. Wende, '89, Buffalo; Franklin W. Barrows, '93, Buffalo; A. W. Bayliss, *ex-officio*, Buffalo; John Parmenter, *ex-officio*, Buffalo.

*Board of Trustees.*—Frank H. Moyer, '72, Moscow, N. Y.; D. A. Currie, '64, Englewood, N. J.; P. W. Van Peyma, '75, Buffalo; William Warren Potter, '59, Buffalo; D. W. Harrington, '71, Buffalo.

On motion the report of the nominating committee was accepted and the secretary instructed to cast one ballot for the candidates named, who were then declared elected.

The program for the day then followed.

*First Paper.*

PROFESSOR HERBERT M. HILL, PH. D.

### GUNPOWDER AND EXPLOSIVES.

Dr. Hill sketched the advances in the manufacture of weapons from the simple forms of the far distant east to the deadly engines of modern warfare, but especially considering gunpowder and explosives.

Gunpowders vary largely in both physical and chemical characteristics with the purposes for which they are designed.

The old-fashioned black powder was a compound of charcoal, sulphur and saltpetre. The latter constituent was added to furnish oxygen for the combustion of the carbon, and the sulphur to add to the volume of gas produced in the process of combustion. It is the propelling power of the gas which sets the bullet in motion. The great disadvantage of the ordinary powder is, that the gas once being freed, and the bullet begins to leave the breech, the pressure becomes less and less owing to the increasing space as the bullet nears the muzzle of the gun, so the volume of gas formed must be very large in order to furnish a high initial velocity at the muzzle. The black powder is unsuitable for warfare because it does not furnish a true gas, but a vapor, and a large amount of unburned carbon to the air which soon renders it impossible to see.

If you should make a simple mixture of the ingredients of black powder and ignite it, the result would be a very slow combustion, creeping along until the whole mass was burned. It has been found necessary to mix the materials very thoroughly in a moist condition and to compress and granulate them before the compound is in any kind of shape to yield a sudden volume of gas when ignited. The glistening surface is given in the last stage of manufacture by treatment with graphite. A sample of black powder was then exploded to illustrate the formation of smoke and residue. A part of the residue consists of potassium sulphate, which readily takes up moisture from the air, so that the inside of the barrel soon becomes wet. This material also readily attacks iron, so if the gun is laid away uncleaned it becomes eroded, and that detracts from its ability to throw a projectile. For blasting purposes this black powder is prepared with a different proportion of ingredients, and the grains are as large as peas.

From the time that guncotton first came to be used in warfare various investigators had in mind the thought that it might be used in place of the black powder. It was known that while it gave most wonderful explosive effects it made no smoke and almost no residue. But the explosive effect was of such terrible character that it could not be used in any ordinary gun. The object then became to manufacture a guncotton which would not have such explosive effect but that it could be adapted to use by ordinary guns. It was found that according to the length of time the cotton was left in contact with the nitric acid the greater or less proportion of nitric acid radicle entered into the compound; that by long treatment a guncotton of such explosive power could be made that the weapon would go to pieces; on the other hand, by treating a shorter time one is produced which has nowhere near the explosive power, burns readily, furnishes gas in sufficient quantity and gives practically no residue. For that purpose the cotton is made into what is known as a colloidal condition, treated with solvents so that it loses its fibrous character and becomes a good deal like glue.

This material is ground in the manufacture of smokeless powder, which leaves it in this finely divided condition. It is also treated with acetone, which granulates it, so at last you have a cellulose nitrate which comes into the market looking a good deal like grains of mustard seed. (Dr. Hill here illustrated the combustion of this variety of powder.) This is relatively a slow burning powder which makes it continue to increase the volume of gas all the time the projectile is inside the gun and until it escapes from the muzzle it is moving faster and faster. Such is the character of the powder used for sporting purposes. For military use we have a powder which has more ability to make gas than the simple nitrate. That effect may be brought about in two ways: either by using a guncotton that has been treated longer with nitric acid, resulting in a product nearer akin to this wonderfully explosive variety used in the torpedo, or by mixing the guncotton with nitroglycerine. The powder now used in many armies contains a large proportion of that substance, the character of the nitroglycerine being largely diluted, so to speak, by the guncotton used with it. Here is a sample of this highly explosive military powder manufactured entirely from guncotton, and differing from the sporting variety in that the cotton has been treated longer with nitric acid, has become correspondingly more unstable, and being more unstable furnishes its volume of gas more quickly. The residue is very light and the smoke not large in amount.

I now exhibit a sample of dynamite, which is about 60 per cent. nitroglycerine absorbed in wood pulp. The high-pressure military powder has about 50 per cent. absorbed in guncotton instead of wood pulp, thereby giving the explosive effect of both. One important point with reference to this powder is the fact that the gases formed are of such character as to have no serious injurious effects on the material of the gun. In manufacturing powder for large guns it is customary to make the individual particles large, and to increase the burning surface to make holes through the larger pieces. (Prof. Hill here exhibited samples of cannon powder.)

In order to produce combustion of this powder it is necessary to have some material which shall carry fire in some manner from the quick stroke of a hammer to the charge. The oldest method was by the actual application of a match or piece of lighted tow to the powder lying in the pan. From that developed successively the flint lock and the use of fulminate of mercury. This was first made into little pills, which were put into the top of the nipple, it being hollowed out for that purpose, and then the sharp point of the hammer striking the pill exploded it. That was the old-fashioned "punch-lock" gun. It was then found advisable to put this fulminate of mercury in a little metal case or "cap," which is used in one form or another for the various kinds of powder.

*Second Paper.*

PROFESSOR WILLIAM H. MOSHER, PHAR. D.

ANCIENT AND MODERN WEAPONS.

In the earliest form of gun using explosive material such as powder, the charge was ignited by a match; but they have become so rare that it was impossible to secure one for this exhibition. Here is the flint-lock, the earliest form we could obtain. It has a tremendous recoil. It was used by both sides during the revolution. Very few of the barrels were rifled. Here is a sample of the flint-lock pistol carried by the officers, whose servant kept them loaded as fast as fired. The ball weighed over an ounce and the amount of shock caused by it was considerable.

The next type, the Remington, very similar to the Springfield, was used during the civil war. It was a percussion lock with a cap lined with fulminate. The cartridges were paper, having the ball in one end and the charge in the other; they were tied with a string, and when loaded the end was bitten off with the teeth and the charge sent home with a ramrod. They produced a very heavy recoil, the ball weighing 482 grains. The gun weighed about nine pounds.

During the civil war various models of semi-breechloading guns were made. Here is one that saw actual service; it breaks at the breech, a paper cartridge was inserted and a cap placed on the nipple. Here is a cavalry carbine carrying the same sized ball. Another model of the same thing was the Gallagher carbine, a forerunner of the modern Maynard. Until the latter years of the war no gun was used requiring a metal cartridge; then the Spencer repeating rifle was used by sharpshooters. Here is a sample, 58 calibre, carrying an ounce ball, rim fire, that is, the fulminate was placed about the rim. The next type was the Springfield breechloader, a few of which were used toward the end of the war. This is the cartridge, known as the 50—70 musket, *i. e.*, a 50 caliber requiring 70 grains of black powder. The next was the 45—70, and following that the 45—75, carrying an ounce ball. The next change in the national weapon was to the Krag-Jorgensen, with a calibre of .30 and using smokeless powder.

In the late war the Spaniards used a Mauser, which is also a bolt gun with a magazine below. The cartridges are fed in bunches or "clips" of five. It has a bore of about .28, and a straight stock.

The same cartridge has been adapted by the Winchester people to a sporting rifle with lever action, the socalled 30—40. This cartridge also comes as the soft-nosed or "dum dum." (The speaker here exhibited the 30—30 Winchester and the Savage and discussed the technical points and mechanism illustrated by the two.)

The chief difference in the manufacture of the old, black powder guns and these using smokeless powder is in the quality of the steel used. The steel used in the 30—40 and the 30—30 requires a tensile strength of over 100,000 pounds to the square inch. There is nineteen tons pressure in the breech of a Krag when the bullet starts. Another difference is in the rifling. By putting in a series of spiral grooves the old smooth barrel is changed into a rifled gun and imparts a rotary motion to the projectile to prevent its turning sideways, or "key-holing." In the muzzle-loader this extends to about two-thirds of the way down; in the breech-loader almost the entire distance.

Dr. Hill and Mr. Mosher then fired full jacketed and dum dum bullets with smokeless powder as well as the lead bullet with black powder, illustrating the difference in penetrating power and sound of report.

*Third Paper.*

WILLIAM H. BERGTOLD, M. D.

EFFECTS OF MISSILES ON BIG GAME.

It has been my privilege during the past eight years to do a good deal of hunting among big game, such as deer, elk and antelope, with an abundance of bear and mountain lion, and among all these I have seen many wounds produced by both the old black powder guns and the modern sporting rifle. The trend of the sporting rifle, from the time of the small "pea" rifle of the old backwoodsman of Kentucky up to ten years ago, has been toward the use of larger balls. The difference in the effect desired in hunting game and in warfare is this: in game we wish to kill, to produce as savage and severe a wound as possible as the first effect; in warfare the object is to produce disability alone, and with as little damage as possible. With the old black powder we found in the West that the tendency was to cling to the large calibre; that the buffalo hunters discarded their Spencers and Sharps, which carried balls weighing an ounce, with a great deal of reluctance. They were accustomed to shoot buffalo at close range and wanted a ball to produce a tremendous wound.

When the buffalo disappeared and left elk, deer and game of that sort, for a number of years the tendency shifted to the use of the Winchester, and the favorite size was 45—70 or 45—90. In 1895 I used a 45—90; in 1896 the Winchesters brought out their first high velocity small calibre sporting rifle.

Now I wish to call your attention to one point, and that is that the modern small calibre rifle in warfare uses a completely jacketed ball. In 1896 I saw the results of using a jacketed ball on game while hunting with a friend who had no soft-nosed ammunition. In three weeks' hunting we didn't have a single opportunity to examine the wound in a deer, elk or antelope, notwithstanding he was a dead shot. I have not the slightest doubt that he hit a great many, and the balls went straight through them.

On the other hand, the effect of the soft-nosed or dum dum bullet on game is startling. I have seen a buck weighing 150 to 300 pounds have the top of his head taken off as completely as though done with a cleaver by a soft-nosed 30—30. I have seen a bull elk at 800 yards drop as though electrocuted, and the post-mortem would show, for example, the ball having entered behind the right shoulder, traversed the lungs and heart and lodged in the shoulder of the opposite side, the lungs and heart being an absolutely pulverised mass as big as my hat. I have seen elk run, 600, 700 or 800 yards, with a black powder, soft lead wound through the heart. The effect on an animal tenacious of life like the mountain lion I saw once. He was about

90 yards away in dense timber. He had stopped to look at us, turning his head back toward his rump. The ball caught him in the mouth, passed out through the occipital bone, pulverising the atlas and neighboring bones. The animal never stirred, except in the twitching of his tail.

We find the small bore, high velocity, soft-nosed bullet is by long odds ahead of any other that has been put upon the market for sporting purposes.

Dr. Bergtold exhibited specimens of soft and full jacketed bullets taken from game post-mortem.

Dr. William Warren Potter, of Buffalo, followed with the subject: Personal experiences in the civil war. His remarks were of a general character, during the course of which he mentioned many incidents showing the destructive nature of the missiles then in use for large and small arms.

Major L. A. La Garde, Surgeon U. S. Army, in charge of Soldiers' Home at Washington, then presented a paper entitled, Gunshot wounds by old and new armaments (see page 873), which was illustrated with the stereopticon.

Lieutenant-Colonel G. Sterling Ryerson, of Toronto, read a paper entitled, My experiences in war—a contrast, 1885-1900, which will be published in a subsequent issue of the JOURNAL. Major William P. Kendall, Surgeon U. S. Army, Fort Porter, Buffalo, read on Experiences in the Philippine war, and Dr. Roswell Park, of Buffalo, discussed the Diagnosis of the location of missiles and treatment.

Dr. William C. Phelps, of Buffalo, read a paper on Experience with gunshot wounds in civil life, which will also be published in a later issue.

The Association then adjourned to dinner at the Iroquois Hotel.

---

CLEFT PALATE.—A. Vander Veer (*Reference Hand-Book of Medical Sciences*) ascribes the cause of this deformity to heredity and the want of a meat diet and of sufficient phosphates on the part of the mother. In confirmation of this view, he cites the fact that several years ago the lions in the Zoological Gardens of London were fed upon flesh containing too large bones for them to break and swallow. The young born while this method of feeding was pursued, had cleft palates and lived but a short time. The lions were then fed upon small animals, whose bones they could break easily, and the young born afterward had perfectly formed palates.—*Denver Medical Times.*

